# Engineering Properties of Superconducting Materials

**DOI:** 10.3390/ma13204652

**Published:** 2020-10-19

**Authors:** Tim Coombs

**Affiliations:** Electrical Engineering Department, Cambridge University, Cambridge CB3 0FA, UK; tac1000@eng.cam.ac.uk

**Keywords:** HTS, bulk superconductors, coated conductors, mathematical modelling, H-formulation

## Abstract

Taking a technology from the laboratory to industry is a long and resource-consuming process. Discovered more than a century ago, the phenomenon of superconductivity is testament to this process. Despite the promise of this technology, currently the only major use of superconductors outside the laboratory is in MRI machines. The advent of high-temperature superconductors in 1986 heralded a new dawn. Machines which do not require cooling with liquid helium are a very attractive target. A myriad range of different superconductors were rapidly discovered over the next decade. This process of discovery continues to this day with, most recently, a whole new class, the pnictides, being discovered in 2006. Many different usages have been identified, including in motors, generators, wind turbines, fault current limiters, and high-current low-loss cables. This Special Issue looks at some of the different factors which will help to realise these devices and thereby bring about a superconducting world

The search for clean energy sources has been a fundamental key in materials research. The development of superconducting materials attracts significant scientific and technological resources towards achieving low costs, as well as suitable and profitable power generation, storage, distribution and transmission. In addition, superconducting electronics can provide devices and circuits with properties not obtainable by any other known technology; i.e., very low-loss, zero frequency-dispersion signal transmission lines, very high-*Q*-value resonators and filters, and quantum limited electromagnetic sensors.

All of these advances require high quality superconducting materials, and, in recent years, great strides have been made to improve the properties of existing materials, as well as the continuing discovery of new systems and materials, such as the pnictides.

In 1911, Heike Kamerlingh Onnes discovered superconductivity in mercury by cooling it down to a frosty 4.2 K (−268.95 °C). Since then, it has been the Holy Grail of material scientists to achieve this transition—from a normal to superconducting state—at room temperature (above 273.15 K or 0 °C). The hope of finding a room-temperature superconductor (RTS) arose after physicists discovered high-temperature superconductivity (HTS) in the 1980s and 1990s in a class of ceramic materials called cuprates [1]. They are characterised by the presence of interleaving copper-oxide layers. Their transition temperature—also known as critical temperature (Tc)—was significantly higher than those of conventional metallic superconductors discovered decades earlier. Even more recently MgB2 and a whole new family based on pnictides [2] were discovered. 

There is a continuous drive towards higher and higher transition temperatures and to date, the highest superconducting Tc achieved, and confirmed, is 203 K, in 2015. From an engineering point of view, although higher transition temperatures are desirable, of greater interest is the development of the engineering properties of the materials. 

The articles in this Special Issue reflect the broad nature of the subject and of the materials available to engineers. Superconductors are available as thin and thick films, single crystals, bulks, and tape. Of this large range, two of the most important to engineers are bulks and tapes. 

Bulks have been available since the very early days of HTS [3]. In a high-quality bulk, the current can flow on the length scale of the superconductor and this property means they can operate as high-field ‘permanent’ magnets [4,5,6]. The field is only limited by the structural properties of the bulks and the source being used to magnetise them. Bulks are typically used in levitation, i.e., bearings or levitated trains. The best bulks have strong pinning properties as the stronger the pinning, the higher the field that they can maintain, and there is a continuous drive towards improved pinning properties.

Tapes, commonly known as coated conductors [7], are more versatile as they can be used to construct coils and hence act as magnets or to carry power at very high power densities. In addition, the property that once the critical current is exceeded they start to develop resistance can both be a blessing in a fault current limiter [8] (where the resistance is desirable and limits the current) and a curse in a magnet where the problem of quenching can cause irreparable damage [9]. Both of these issues are covered in this Special Issue.

The final topic covered in this Special Issue highlights the enormous effort which has been devoted to predicting the behaviour of superconductors by ever more sophisticated modelling techniques [10].
